# Supplementary material for: HR+/HER2– Advanced Breast Cancer Treatment in the First-Line Setting: Expert Review
Source: Curr Oncol. 2023 Jun 2;30(6):5425–47. doi: 10.3390/curroncol30060411 (PMC10297170; doi:10.3390/curroncol30060411)
Supplement: Supplementary file 1 [file curroncol-30-00411-s001.zip › curroncol-2393013-supplementary.pdf]

Supplementary Table S1. Dose Modification for Specific Adverse Reactions Listed in at Least One CDK4/6i Product Monograph

	Grade 1	Grade 2	Grade 3	Grade 4
**Neutropenia**	ANC 1000/mm^3 –<LLN<br>No modification	ANC 500/mm^3–<1000/mm^3<br>Interrupt until Grade ≤2 and resume at same dose<br>*Recurrent or febrile neutropenia*: Interrupt until Grade ≤2 and resume at next lower dose<br>PAL: As above if Day 1 of cycle; if Day 15 of first 2 cycles, continue at current dose and adjust based on CBC repeated Day 22	ANC <500/mm^3<br>Interrupt until Grade ≤2 and resume at next lower dose	
**ALT/AST elevations without increase in total bilirubin >2x ULN**<br>No specific PAL recommendations	ALT/AST >ULN–3x ULN<br>No modification	ALT/AST >3x–5x ULN<br>RIB: if BL <Grade 2, interrupt until recover to BL, then resume at same dose<br>If BL Grade 2, no modification<br>ABE: No modification<br>*Recurrent*: Interrupt until ≤BL (ABE, RIB) or Grade 1 (ABE) and resume at next lower dose	ALT/AST >5–20x ULN<br>Interrupt until ≤BL (ABE, RIB) or Grade 1 (ABE) and resume at next lower dose	ALT/AST >20x ULN<br>Discontinue
**Total bilirubin >2 x ULN**<br>No specific PAL recommendations	ALT/AST >3x ULN<br>Discontinue			
**QTcF prolongation + electrolytes**<br>No specific PAL or ABE recommendations		QTcF >480 ms or recurrent ≥481 ms<br>Interrupt until <481 ms then resume at next lower dose<br>Correct serum electrolytes before resuming	QTcF >500 ms<br>Interrupt until <481 ms then resume at next lower dose<br>Correct serum electrolytes before resuming	Torsade de Pointes; polymorphic ventricular tachycardia; unexplained syncope; signs / symptoms of serious arrhythmia<br>Permanently discontinue
**Diarrhea**<br>No specific RIB or PAL recommendations	Increase of <4 stools/day over BL<br>No modification	Increase of 4–6 stools/day over BL<br>Does not resolve within 24 hours to ≤Grade 1: suspend until resolution; no modification<br>*Persistent / recurrent after resuming despite maximal supportive measures:* suspend until ≤Grade 1; resume at next lower dose	≥7 stools/day over BL; hospitalization; life-threatening consequences<br>Suspend until ≤Grade 1; resume at next lower dose	
**ILD / pneumonitis**	Grade 1 (asymptomatic)<br>No modification*<br>No specific PAL recommendations	Grade 2 (symptomatic)<br>No modification<br>*Persistent (7 days) / recurrent despite maximal supportive measures:* suspend until BL (ABE) or ≤Grade 1 (ABE, RIB) and resume at next lower dose[†]<br>No specific PAL recommendations	Grade 3 or 4 (severe)<br>Discontinue	
**VTE (advanced breast cancer)**<br>No specific RIB or PAL recommendations	Grade 1<br>No modification	Grade 2<br>No modification	Grade 3 or 4<br>Suspend dose and treat as clinically indicated; resume when patient is clinically stable	

*The ribociclib product monograph recommends initiating appropriate medical therapy and monitoring as clinically indicated.

[†]The ribociclib product monograph recommends performing an individualized benefit-risk assessment when considering resuming.
